# Comments on “Effect of remimazolam besylate vs propofol on incidence of postoperative delirium in older patients undergoing hip surgery: a randomized noninferiority trial”

**DOI:** 10.1097/JS9.0000000000002454

**Published:** 2025-07-23

**Authors:** Wenwen Zheng, Xiaocou Wang, Xuefei Ye

**Affiliations:** The Second Affiliated Hospital and Yuying Children’s Hospital of Wenzhou Medical University, Wenzhou, Zhejiang, People’s Republic of China

*Dear Editor*,

We read with interest the article by Fang *et al*, which concluded that among older adults undergoing hip surgery, general anesthesia with remimazolam besylate was not inferior to propofol with regard to the incidence of POD or adverse events^[1]^. However, we have several concerns about this trial. We hope that clarification of these concerns could enhance the interpretation of the study and guide future research efforts.

First, the trial did not specify whether anticholinergic agents, which are commonly used during clinical anaesthesia, were used intraoperatively. These agents may have been used to reduce secretions, in combination with neostigmine and glycopyrrolate or atropine to reverse neuromuscular blockade, or for bradycardia management with atropine. Anticholinergic agents, by blocking central and peripheral cholinergic receptors, may lead to decreased acetylcholine levels. Impaired cholinergic neurotransmission is one of the key pathophysiological mechanisms of postoperative delirium and may directly increase the risk of delirium^[2]^. In elderly patients with diminished hepatic and renal function, the duration of action of anticholinergic agents may be prolonged, thereby exacerbating the cumulative effects on the central nervous system. If there were differences in the frequency or dosage of anticholinergic agents between the two groups, this could represent an uncontrolled confounding factor.

Second, the study did not compare the postoperative pain levels between the two groups of patients, nor did it mention the use of analgesics. Analgesics may include opioids, nonsteroidal anti-inflammatory drugs, other types of analgesics, and the use of patient-controlled analgesia pumps. Effective postoperative acute pain management can reduce the incidence of postoperative delirium, with postoperative pain being one of the independent risk factors for delirium^[3]^. Although the ilioinguinal fascia block was intended to standardize postoperative analgesia, the lack of pain data may limit the comprehensive interpretation of the results.

Third, it is unclear whether there were differences in the postoperative urinary catheterization status between the two groups of patients. Pain or discomfort from urinary catheterization can activate the stress response, leading to sympathetic nervous system excitation and the release of inflammatory cytokines, which may induce delirium^[3]^. If there were differences in catheterization between the two groups, this could impact the results.

Finally, the study only provided a general comparison of blood loss but did not describe transfusion-related events. Perioperative blood transfusions^[4]^, especially platelet transfusions^[5]^, are considered independent risk factors for postoperative delirium. However, this clinical trial did not report on transfusion status.

## Data Availability

Data sharing is not applicable to this article.
